# Interactive patient education via an audience response system in cardiac rehabilitation

**DOI:** 10.1177/2050312120942118

**Published:** 2020-08-25

**Authors:** Dietrich Stoevesandt, Andreas Weber, Andreas Wienke, Steffi Bethge, Viktoria Heinze, Simone Kowoll, Axel Schlitt

**Affiliations:** 1 Department and Outpatient Clinic for Radiology, University Hospital Halle (Saale), Halle (Saale), Germany; 2 Institute for Health and Nursing Science, Martin-Luther-University Halle-Wittenberg, Halle (Saale), Germany; 3 Institute of Medical Epidemiology, Biometry, and Information Technology, University Hospital Halle (Saale), Halle (Saale), Germany; 4 Paracelsus-Harz-Klinik Bad Suderode, Quedlinburg, Germany; 5 Coordination Center for Clinical Studies, Medical Faculty, Martin-Luther-University Halle-Wittenberg, Halle (Saale), Germany; 6 Medical Faculty, Martin-Luther-University Halle-Wittenberg, Halle (Saale), Germany

**Keywords:** Cardiac rehabilitation, patient education, audience response system, questionnaire

## Abstract

**Objectives:**

Patient education and compliance play an important role in the success of rehabilitation in cardiovascular diseases. The aim of this study is to analyze whether interactive learning methods, in this study, the audience response system with a “clicker,” can improve the learning success of patients during and after their rehabilitation process.

**Methods:**

In a randomized, prospective cohort study, a total of 260 patients were randomized to either an interactive training group using Athens audience response system or to a control group without the use of audience response system during the educational sessions. Patients were taught and tested on four different topics concerning their primary disease: heart failure, arterial hypertension, prevention of cardiovascular diseases, and coronary heart disease. After each session, the patients had to answer questions on the previously taught topics via questionnaires. These questions were asked again at the day of discharge, as well as 3 and 12 months after discharge. Additional information on the patients’ health, plus their mental status, was gathered with the help of further questionnaires (HADS and SF-12).

**Results:**

A total of 260 patients (201 men and 59 women) were recruited. The patients were on average 61.1 ± 11 years old. A significant short-term effect on the patients’ knowledge about their disease was found immediately after the educational sessions in the intervention group. However, there was no long-term effect in either the intervention or control group. Although there was no statistical significance found in any of the observations, a positive short-term effect on learning capacity as well as positive trends in mental and physical health after discharge could be found in patients after the use of audience response system during their rehabilitation.

**Conclusion:**

This study provides interesting and new data on the use of an interactive learning method for patients to gain knowledge about their primary disease and eventually improve their physical and mental health status in a long-term perspective. By implementing different and new ways of teaching and interaction during the hospitalization, not only patients, but also medical staff and caregivers could benefit.

## Introduction

Cardiovascular diseases are still the leading cause of death in Germany according to the German Federal Statistical Office. The coronary heart disease (CHD), as one particular disease of the pathology, is associated with the highest mortality.^1^ By reducing modifiable risk factors and containing the progression of cardiovascular disease, mortality can be lowered and patients’ quality of life improved. Primary, secondary, and tertiary prevention aims to achieve these goals starting with the detection and avoidance of risk factors like obesity, smoking, and diet, to then recommending certain lifestyle changes like blood pressure control, stress management, and healthy, physical activity. Especially, cardiac rehabilitation has proven to be effective in terms of tertiary prevention in patients with CHD.^2^ A new approach on patient education may positively influence the success of the rehabilitation. This work studies the interactive patient education with an audience response system (ARS) to analyze the connection *between a more interactive educational tool and a knowledge-based coping eventually improving patients’ outcome.*

### Patient education sessions

In the 1980s, a new approach on how patients can cope with their disease and how this may improve their conditions arose. Soon, multiple studies on patient education were conducted internationally.^3,4^ Nowadays, specialized clinics and centers offer specific instructions for patients and medical staff:

Patient education sessions are interactive group programs for people with primarily chronic disorders. They are designed to improve the cooperation (compliance) of patients with their medical treatment and increase their ability to deal with their disease independently (self-management) in cooperation with professional support.^5^

According to the studies on patient education available to date, there are many different results concerning the practicability and efficiency of given subject. A review article including 13 randomized controlled studies investigated the education of patients with CHD as a primary intervention. Patient education consisted of individual or group educational sessions, telephone conferences, Internet-based methods, and a follow-up of at least 6 months. In summary, no significant differences were seen in terms of mortality, morbidity, and quality of life between the intervention group (IG) and the control group (CG).^6^

In another study, Reusch et al.^7^ compared patients who underwent an intervention consisting of an interactive, patient-oriented education program with a CG that received lecture-based education only. The objective was to analyze changes in motivation and self-reported behaviors in the domains of sports, diet, and relaxation. The intervention contained short talks, group discussions, and individual tasks based on a health education program compiled by the German pension insurance. Although no significant differences in motivational changes due to patient education could be confirmed, the IG showed more advanced motivation regarding diet and sports on multiple times during the follow-up.

Another study investigated the effect of computer-based patient education compared to the formerly used tutorial learning. The study group found significant main group effect on the exercise knowledge in the post-training and follow-up phase. In addition, patients preferred the computer-assisted learning method as they were able to study at their own pace and repeat difficult content.^8^ Another example for the use of media in patient education was demonstrated.^9^ They found positive effects of telephone follow-up in a randomized controlled study in Northern Germany. Patients showed a lowered Framingham Risk Score after receiving monthly health instructions by instructed nurses over the course of a year.

The previously shown various results suggest that although patient education may improve motivation to change health behaviors, the most effective method has not yet been found. Whether inpatient education, computer-assisted tools, or outpatient media-based communication is superior remains unclear. Furthermore, the evaluation of new educational methods in cardiac rehabilitation is still an issue and basis of this study. The use of an ARS could be of potential benefit in this regard. The implementation of this easy, interactive educational method during rehabilitation shows potential for establishing a long-lasting learning tool to improve patient knowledge and compliance.

### ARS

In the 1960s, the first ARS was developed in the United States as a supportive teaching method in schools. From 1985 it was used under the name “Classtalk I” for the purposes of interactive teaching and learning among students.^10^ Technologically high-quality and easy-to-use ARS have been available at a low cost since 1999, and thus went on to gain broad international acceptance after the turn of the millennium. Although designing effective and inspiring questions can be difficult,^11^ ARS have already been tested and used with multiple positive results in a large number of universities offering a variety of degree courses, as well as in schools.^12^

Using an ARS makes it possible to instantly analyze participants’ level of knowledge during a talk or presentation. Each participant is given a response card or “clicker,” similar to a calculator, which enables them to enter their preferred response to questions presented in a multiple choice format. Answers are sent to the response receiver via an infrared signal, a radiofrequency signal (RE-modulated), or via an Internet connection. This wireless application, combined with the appropriate software, offers a simple way to graphically display answers and visualize response behavior immediately. Furthermore, participants essentially preserve their anonymity during the intervention. Thus, participants experience no embarrassment from not knowing the correct answers right away and frequently unanswered or difficult questions are given a forum for discussion.^13^

To date, however, no studies have been performed investigating the benefit of an ARS used by patients with cardiovascular diseases in a rehabilitation center. The aim of this study is to analyze whether ARS can improve personal reflection, sharpen concentration, and cause a higher level of understanding for the patients disease in a clinical setting during the rehabilitation process. In addition, the regular use of an ARS may achieve better cognition and stimulate discussions among participating patients leading to positive long-term effects such as implementing a healthy lifestyle.

### Potential influencing factors on learning

Disease and hospitalization often correlate with the development of psychological problems and a decline in patients’ quality of life. In their study, Polat et al.^14^ show how depression levels decreased and how quality of life increased over the course of their treatment of colorectal cancer measured by different questionnaires. Patients were intensely informed about their disease and the planned therapy before and during their treatment to improve coping strategies and lower disease-related depression over the time of therapy. In this study, the question arose whether enhanced patient education may also show changes in these factors. Thus, two additional questionnaires came to use during the education and rehabilitation process: the hospital anxiety and depression scale, German version (HADS-D) questionnaire to detect disease-related signs of depression and anxiety^15^ and the short-form health survey (SF-12) SF-12 questionnaire to evaluate the current quality of life and limitations due to the disease.^16^

## Methods

### Study design and setting

This study is a randomized, prospective cohort study supported by the German Heart Foundation (ClinicalTrials ID Nr. NCT02185391). The main goal is to compare two different approaches on patient education during rehabilitation for cardiovascular disease. The patients were randomly assigned to either an IG, which received interactive education using an ARS, or to a CG, which received patient education without an ARS. In the end of each educational session, patients had to fill out a questionnaire on the topic recently presented. The two groups analyzed over the course of 12–13 months (Figure 5).

### Sampling and recruitment

The study population consisted only of patients at the Paracelsus-Harz-Clinic, Quedlinburg, Germany, who received rehabilitation for their cardiovascular disease. A total of 130 patients undergoing rehabilitation were initially recruited to both the IG and the CG (n = 260). This defined sample size ensured that criteria of sample size determination calculated with the G-Power program were fulfilled. Based on a planned study power of 95%, this calculation (α = 0.05), at a drop-out rate of 25%, yielded a sample size of at least 100 subjects per group at the outset of the study (G-Power calculation with the following assumptions: analysis of variance (ANOVA), repeated measures, t = 4; effect size f = 0.5; correlation between measuring times, corr = 0.5). The randomization and selection in either the IG or CG depended on the different days of admission during the week. Patients coming to the clinic on Mondays, Wednesdays, or Fridays were assigned to the IG using ARS during their educational sessions. Patients admitted on Tuesdays and Thursdays were assigned to the CG.

All patients had to match two inclusion criteria for the study. First, they had to be of age ⩾18 years and second, the reason for the inpatient stay had to be a cardiac rehabilitation in the Paracelsus-Harz-Clinic Bad Suderode, Quedlinburg. Patients could not be included, if they met one of the following exclusion criteria: preexisting diseases or functional disorders which, according to the investigator’s assessment, precluded the participation in the clinical trial (dementia, psychotic disorders, drug, or alcohol dependency, etc.), missing declaration of consent, or a simultaneous participation in another clinical trial.

After receiving approval from the ethics committee of the Medical Faculty of the Martin Luther University Halle-Wittenberg (clinical ID 2013-125), Germany, and written consent from the patients, baseline parameters, and general patient information were assembled (see Table 1). Socio-demographic and other disease-relevant parameters like preexisting comorbidities were determined according to clinical standards. Data on dietary habits, physical activity, and disease course were collected using a study-specific questionnaire. Medical parameters such as an initial echocardiogram, blood pressure, weight, body mass index (BMI), waist-to-hip ratio (WHR), and laboratory parameters like blood glucose and total cholesterol were tested prior to the intervention process and rehabilitation. The current quality of life and health status was evaluated using the SF-12 questionnaire, where patients answer 12 questions on current life situations or health restrictions concerning limitations in their physical or mental status, social activities, bodily pain, or vitality. Anxiety and depression were evaluated using the HADS-D questionnaire by answering both seven questions on anxiety or depression-related items (see Supplemental Appendix for questionnaires).

**Table 1.** Baseline variables.

Variable	Total (n = 260)	IG (n = 130)	CG (n = 130)	*p* value
Age (years)	61.1 ± 11	61.7 ± 11	60.6 ± 11	0.410
Gender				0.459
Male	77.3	79.2	75.4	
Female	22.7	20.8	24.6	
Body mass index (kg/m^2^)				
Admission	29.3 ± 5.2	29.4 ± 5.5	29.2 ± 4.9	0.827
Discharge	29.1 ± 4.9	29.1 ± 5.2	29.1 ± 4.7	0.956
Smokers (%)	14.2	12.3	16.1	0.375
Coronary heart disease (CHD) (%)	93.5	90.8	96.2	0.079
Hypertension (%)	87.3	86.2	88.5	0.464
Dyslipoproteinemia (%)	87.7	87.7	87.7	1.000
Diabetes mellitus (%)	30.8	31.5	30	0.788
Positive family history for CHD (%)	45.4	47.7	43.1	0.455
Acute myocardial infarction (%)	89.2	86.2	92.3	0.109
Atrial fibrillation (%)	9.6	6.9	12.3	0.141
Kidney failure (%)	14.2	11.5	16.9	0.241
Pulmonary embolism (%)	0.8	0	1.5	0.156
COPD				0.736
Bronchial asthma	1.9	2.3	1.5	
COPD	6.2	6.2	6.2	
Other lung diseases	3.5	4.6	2.3	
Educational level (%)				0.612
No qualification obtained	2.3	3.1	1.5	
Apprenticeship	76.9	77.7	76.2	
Degree course	20.8	19.2	22.3	
Employment (%)				0.602
Employed	48.5	46.9	50	
Unemployed	10	11.5	8.5	
Retired	44.2	43.1	45.4	
Length of inpatient stay, days	22.7 ± 3.7	22.3 ± 4	23 ± 3.3	0.113

IG: intervention group; CG: control group; COPD: chronic obstructive pulmonary disease.

Continuous variables are given as mean value ± standard deviation, categorical variables in percentages.

### Intervention

The staff providing the patient education was instructed on a standardized teaching process. A manual for patient education was compiled, defining the basic parameters “target group,” “group size,” “objective,” “content of patient education,” and “methodological approach,” thereby ensuring quality and similarity during the process. Each patient of the IG was handed a “clicker” at the beginning of the session. Using the ARS, patients of the IG were actively involved in the educational process. Patients of the IG had to answer four questions on the content during the talk via ARS. These questions consisted of subject matters previously discussed during the session and, as such, could be answered correctly if participants paid sufficient attention. Patients of the CG did not receive an ARS system. They attended the same sessions information-wise and listened to the talk with identical content and standardized way of information delivery. In contrast to the IG, those patients finished each learning session without any questions asked or discussed during the session.

### Data collection and analysis

This study consisted of patients spending 3 weeks at the center, in order to improve their health and complete a standardized rehabilitation program. Most of the patients suffered not only a cardiovascular disease, but also concomitant diseases such as diabetes mellitus or chronic obstructive pulmonary disease (COPD). During their 3 weeks, the study provided up to eight talks possible to attend on following subject matters: prevention of cardiovascular diseases, heart failure, arterial hypertension, and CHD. The basic, further educational measures during inpatient treatment were adhered to for both the IG and the CG in line with clinical standards. Following the session, participants of both groups were given a standardized questionnaire on the content of the talk. Each questionnaire consisted of 10 statements that had been made during the talk and which could be answered with “correct,” “incorrect,” or “I don’t know..” A maximum score of 10 points could be reached, on the basis of which the learning effect was measured over time. To avoid repetitions in the questions, the wording used for the questions during the sessions with the ARS did not correspond to the questions in the final questionnaires.

At the end of the rehabilitation period (after 3 weeks), medical parameters were measured a second time and each subject was given a concluding, combined questionnaire on the whole content consisting of the four talks. Questions containing similar content were excluded, so a total of 30 questions were to be answered in this merged final questionnaire. In addition, the SF-12 and HADS-D questionnaires were filled out once again to evaluate any changes in their self-perception on quality of life and depression or anxiety due to the primary disease. A planned follow-up period of 12 months was achieved in 100% of the patients. The combined questionnaires with questions on the four topics during the educational sessions were sent out to patients again after 3 and 12 months following their discharge. A total of 30 questions were to be answered and sent back by the patients.

The collected data were manually transferred to Excel and analyzed as follows: continuous variables were described as mean and standard deviation; skewed variables as median, 25% and 75% quartiles. Categorical variables were documented as a percentage. For comparison of metric, normally distributed variables, a t-test was used. The Mann–Whitney U test was used to compare skewed variables. For normally distributed, categorical variables, the chi-square test was employed. The p values <0.05 were considered significant. Statistical analyses were performed using SPSS statistics (SPSS Inc, Chicago, IL, USA) software.

## Results

### Baseline variables

The study completed the recruitment of 260 subjects (201 men and 59 women) in October 2014 and follow-up was concluded in October 2015 for all 260 patients. The mean age of the patients was 61.1 years. More than 75% were male, and the mean BMI was 29.3 kg/m^2^. The vast majority of subjects were undergoing rehabilitation due to CHD. Table 1 shows the comparison between the groups in terms of the following baseline variables: gender, weight, risk factors (smoking, dyslipoproteinemia) comorbidities, education, employment status, and length of inpatient stay. The data in the table show that there are no major differences when comparing baseline variables of the two groups. However, CHD (96.2% vs 90.8%), kidney failure (16.9% vs 11.5%), and the presence of atrial fibrillation (12.3% vs 6.9%) were numerically more common in the CG. Regarding the “educational level,” it becomes clear that the majority of subjects in both the IG and the CG had completed an apprenticeship. The proportion of university graduates and those with no educational qualification jointly accounted for approximately a third. In contrast, the percentage of unemployed subjects was extremely low, while the percentages of employed and retired subjects differed by only a few percentage points. A significant difference was seen only for the variable “transfer to an acute unit during hospital stay” (1.5% IG vs 6.9% CG, p = 0.031). Medication and laboratory values were typical for a patient group with cardiovascular disease (particularly CHD) following an acute event, whereby there was no significant difference between these parameters in the IG and the CG at any measurement point.

### Learning effect of ARS

This prospective study on two well-balanced randomized groups of cardiovascular patients shows that using an ARS can achieve short-term learning success in patient education sessions and, thus, the use of an ARS during such sessions promotes patients’ concentration and capacity to absorb information. However, this effect was no longer visible at the time of discharge from the rehabilitation center, or after 3 and 12 months.

**Figure 1.**
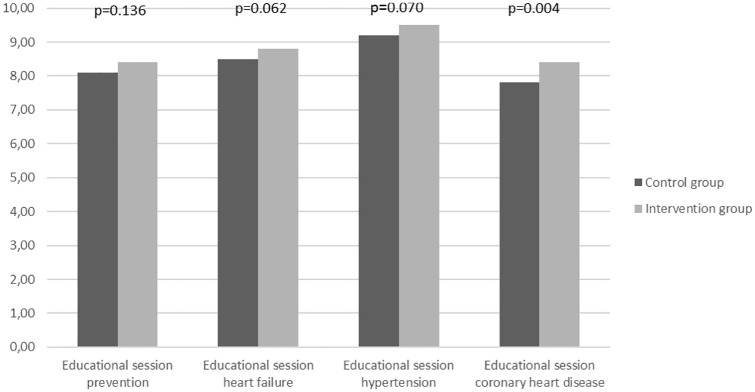
Results for the four different questionnaires during inpatient stay (maximum 10 points each).

Figure 1 shows the results of the four different questionnaires during the inpatient stay. A total maximum of 10 points was achievable by both the IG (ARS) and the CG. Patients of both groups performed comparatively well with a mean score of 8.5 right answers. Nonetheless, the graphic shows slightly better short-term results in the IG with one statistically significant and two trending results (heart failure talk: 8.8 ± 1.2 vs 8.5 ± 1.4, p = 0.062; arterial hypertension talk: 9.5 ± 0.8 vs 9.2 ± 1.3, p = 0.070; cardiovascular disease prevention talk: 8.4 ± 1.4 vs 8.1 ± 1.6, p = 0.136; CHD talk: 8.4 ± 1.5 vs 7.8 ± 1.4, p = 0.004).

**Figure 2.**
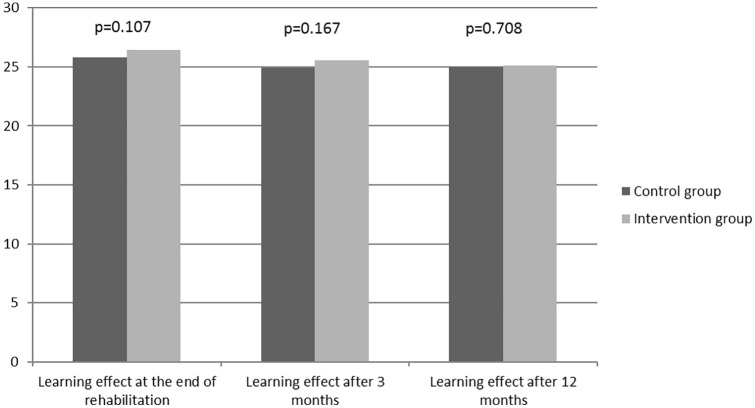
Results of the combined questionnaire at the time of discharge, after 3 and 12 months (maximum 30 points each).

Figure 2 presents the results for the combined questionnaire containing 30 questions on the four different educational sessions during the patients’ rehabilitation. This combined questionnaire was handed out at three different times: on the day of discharge, after 3 and 12 months. Unlike in Figure 1, there was no statistical significant difference found regarding the number of right answers between the two groups at any point. An average of 25 right answers out of 30 questions was noticed in both the IG and the CG. Furthermore, there was no change in these numbers after 3 and 12 months. The initially assumed trend toward a better learning effect using ARS in Figure 1 was decreasing over the observation period.

### Results of the HADS-D and SF-12 questionnaires

**Figure 3.**
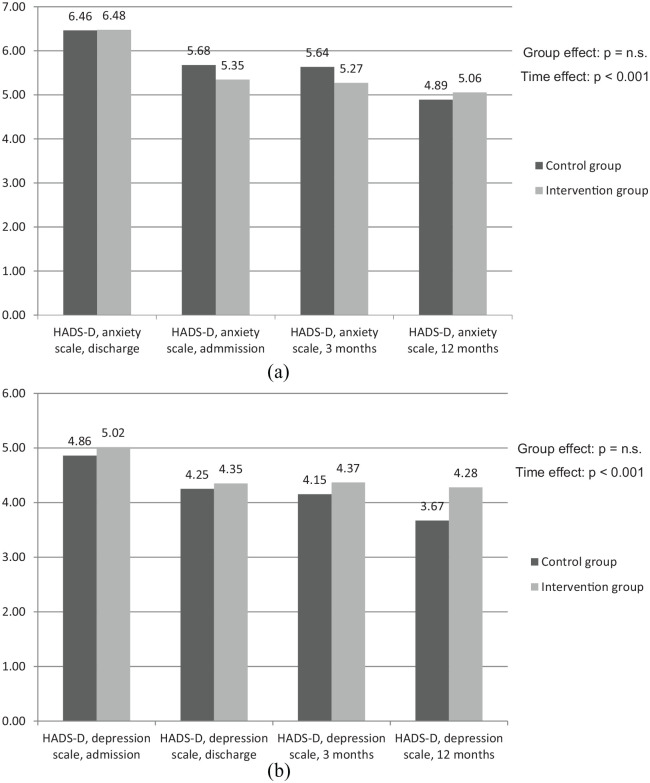
Points in HADS-D questionnaire: (a) anxiety and (b) depression.

Figure 3(a) and (b) shows the results of the HADS-D questionnaires. In general, patients of both the IG and the CG show very similar scores regarding anxiety and depression levels (HADS anxiety at discharge: IG 6.48 points vs CG 6.46 points). In comparison of the two different contents (anxiety and depression), higher scores in anxiety-related issues occurred. With regard to changes over time, it can be stated that signs of both anomalies, anxiety and depression, were decreasing over time (depression on admission IG 5.02 and CG 4.86 vs IG 4.28 and CG 3.67 after 12 months).

**Figure 4.**
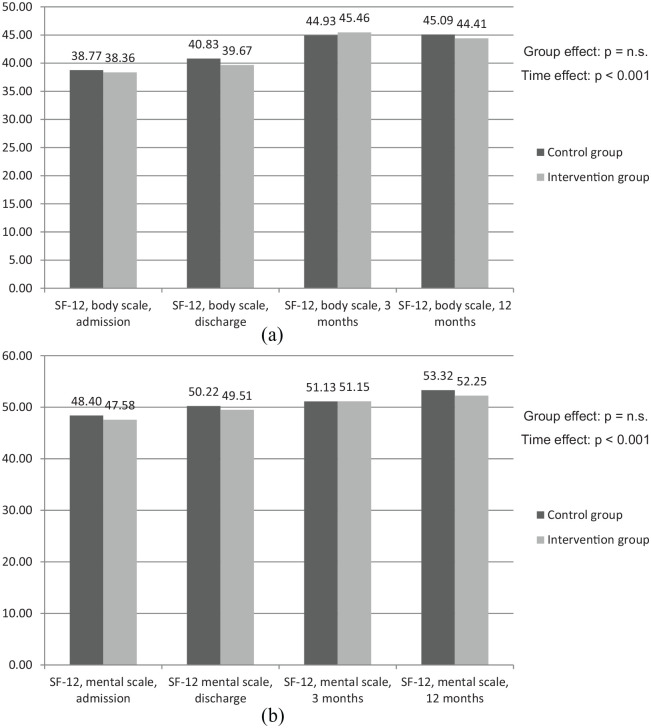
Points in SF-12 questionnaire: (a) physical scales and (b) mental scales.

Figure 4(a) and (b) shows the results of the SF-12 questionnaires reporting on current quality of life regarding mental and physical status. As in the HADS questionnaire, patients of both the IG and the CG show very similar scores when comparing the two groups (SF-12 physical scale at admission: IG 38.68 points vs CG 38.77 points). In comparison of the two different measures (physical and mental status), higher scores in the mental scales occurred. With regard to changes over time, it can be stated that both factors, physical and mental quality of life, were increasing over time (mental capacity on admission IG 47.58 and CG 48.40 points vs IG 52.25 and CG 53.32 points after 12 months).

**Figure 5.**
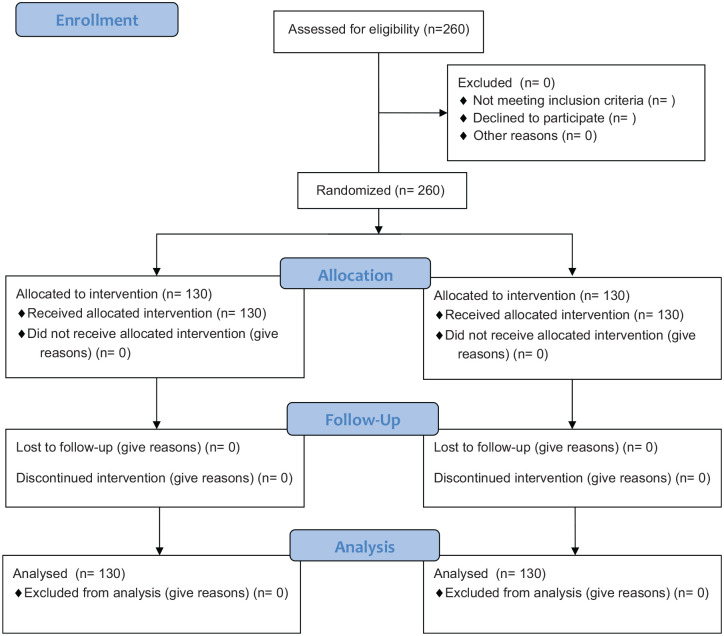
CONSORT 2010 flow diagram.

Although a positive time effect is demonstrated for all parameters observed in both groups, there was no statistical significance in differences between the IG and the CG in terms of these parameters at any of the time points investigated. At most, these results can be interpreted as a trend showing an improved physical and mental health status after the discharge in general.

## Discussion

### Learning effect of ARS

This study presents a valuable contribution to the existing study situation on patient education and rehabilitation as there has never been used an ARS in this inpatient and rehabilitation setting before. The results of this study give an interesting insight on how intensified teaching methods during the hospitalization can improve patients’ health in multiple ways. The data in this study show a short-term effect, but no medium- or long-term effect on the learning success in patients undergoing cardiac rehabilitation and simultaneously using ARS during the educational sessions. This effect has also been found by Tregonning et al.^17^ The study group used the ARS during their lectures on obstetrics and gynecology for medical students at The University of Western Australia. Their data showed a statistically significant improvement on the immediate post-lecture quizzes, but no long-term effect when retesting the students 5 weeks after the lecture.

However, other study groups present different data on this subject. Contrary to this study’s results, Rubio et al.^18^ showed that long-term learning effects can be achieved in a clinical working environment as a result of using an ARS. The authors used ARS during the teaching of their own residents. Employees receiving interactive teaching sessions via ARS showed significantly higher learning and long-term retention scores on the post-lecture tests on the day of the lecture and 3 months later.

Miller and Hartung^19^ presented an even more general approach on the use of ARS in the hospital environment. Using the interactive teaching and learning method on rehabilitation nurses, medical staff, patients, and even caregivers of patients, an increase in motivation and informed decision-making was found. Thus, more studies with a wider usage of interactive teaching and learning methods should follow this study to strengthen the evidence of given methods and create more recommendations on how to improve the dealing with diseases during therapy and after discharge.

### Potential influencing factors on learning

This study showed similar outcomes between the IG and CG regarding the comparison of physical and mental health achieved with the SF-12 and HADS-D questionnaires. However, over the course of the study, a trend of improvement was seen in all parameters investigated. This prompts the question of whether this is an effect of rehabilitation, the interactive educational sessions, or of the natural course in cardiac patients after being discharged from the hospital. A recent US study on almost 5000 myocardial infarction patients demonstrated the same positive course over a period of 12 months, irrespective of participation in cardiac rehabilitation.^20^ However, it should be critically noted that the procedure in cardiac rehabilitation in the United States, particularly due to its unimodal, activity-oriented approach, differs from the multimodal rehabilitation approach usual in German-speaking countries. Furthermore, a more informed and knowledgeable patient may show better coping strategies.

Nonetheless, the question arises if there are any factors that show a negative influence on learning success and patients’ compliance. Factors that potentially reduce the learning capacity and mental flexibility to cope with a disease, such as preexisting or disease-related psychological issues, multiple comorbidities, and advanced age, come to mind. Depression or anxiety induced by a severe diagnosis, in particular, can negatively influence the course of treatment and follow-up care. In their study, Polat et al.^14^ show how depression levels decreased after patients were intensely informed about their disease and the planned therapy over the course of their treatment. More studies and data are needed to gain more insight on such relations and should be investigated in the future. Another study, by a German study group, found that patients in cardiac rehabilitation show a comparatively high occurrence of cognitive impairment. A reduced capacity to learn and cope with their disease was found not only in the elderly, but also in younger patients.^21^

### Limitations of the study

Although this study provides important results, there are few limitations to be noted. A small sample size of the two patient groups may limit the power of the results. Thus, the data may not be considered representative and cannot be generalized to a larger population. The chance of over- or underestimation of results is often linked to small samples sizes. In addition, most results show no statistical significance, but rather trend statements. Second, the data derive from only one clinical center. Thus, the clinical practice heavily relies on the local practitioners and patients.

Considering the overall high performance of patients both in the IG and the CG, the questions content may have been too easy. We cannot exclude the possibility of patients “cheating” during the ARS sessions by exchanging opinions and information with other participants. Also, follow-up questionnaires after 3 and 12 months may have been answered with help of relatives, the Internet, or other sources.

Moreover, questionnaires used in this study were not validated although pilot-tested in a small study with 10 patients, which is another limitation of the study.

### Conclusion, recommendations, and outlook

Considering the various data and different results on ARS in general, in addition to a rather weak study situation regarding the use of ARS on patients, this article can only make assumptions and show trends on how interactive teaching can influence the patients’ health. Although there was no statistical significance found in any of the observations, a positive short-term effect on learning capacity as well as positive trends in mental and physical health after discharge could be found in patients after the use of ARS during their rehabilitation.

## Supplemental Material

all_questionnaires_150 – Supplemental material for Interactive patient education via an audience response system in cardiac rehabilitation Click here for additional data file. Supplemental material, all_questionnaires_150 for Interactive patient education via an audience response system in cardiac rehabilitation by Dietrich Stoevesandt, Andreas Weber, Andreas Wienke, Steffi Bethge, Viktoria Heinze, Simone Kowoll and Axel Schlitt in SAGE Open Medicine

CONSORT_Checklist – Supplemental material for Interactive patient education via an audience response system in cardiac rehabilitation Click here for additional data file. Supplemental material, CONSORT_Checklist for Interactive patient education via an audience response system in cardiac rehabilitation by Dietrich Stoevesandt, Andreas Weber, Andreas Wienke, Steffi Bethge, Viktoria Heinze, Simone Kowoll and Axel Schlitt in SAGE Open Medicine

Supp_material_Bestion_et_al._review – Supplemental material for Interactive patient education via an audience response system in cardiac rehabilitation Click here for additional data file. Supplemental material, Supp_material_Bestion_et_al._review for Interactive patient education via an audience response system in cardiac rehabilitation by Dietrich Stoevesandt, Andreas Weber, Andreas Wienke, Steffi Bethge, Viktoria Heinze, Simone Kowoll and Axel Schlitt in SAGE Open Medicine

## References

[1] Stang A Stang M An inter-state comparison of cardiovascular risk factors in Germany: towards an explanation of high ischemic heart disease mortality in Saxony-Anhalt. Dtsch Arztebl Int 2014; 111: 530–536. 25145511 10.3238/arztebl.2014.0530 PMC4148713

[2] Rauch B Davos CH Doherty P, et al The prognostic effect of cardiac rehabilitation in the era of acute revascularisation and statin therapy: a systematic review and meta-analysis of randomized and non-randomized studies: the cardiac rehabilitation outcome study (CROS). Eur J Prev Cardiol 2016; 23: 1914–1939. 27777324 10.1177/2047487316671181 PMC5119625

[3] Owens JF McCann CS Hutelmyer CM. Cardiac rehabilitation: a patient education program. Nurs Res 1978; 273: 148–150. 248184

[4] Mampuya WM. Cardiac rehabilitation past, present and future: an overview. Cardiovas Diagnos Therap 2012; 2(1): 38–49. 10.3978/j.issn.2223-3652.2012.01.02 PMC3839175 24282695

[5] Zentrum Patientenschulung. Indikationsübergreifende Beschreibungs- und Bewertungskriterien für Patienten-schulungen—Ergebnisse des Delphi-Verfahrens, 2006, http://www.zentrum-patientenschulung.de/theorie/schulungskonzept/

[6] Brown JP Clark AM Dalal H, et al Patient education in the contemporary management of coronary heart disease. Cochrane Database Syst Rev 2010; 12: CD008895. 10.1002/14651858.CD008895 PMC4176666 25267909

[7] Reusch A Ströbl V Ellgring H, et al Effectiveness of small-group interactive education vs. lecture-based information-only programs on motivation to change and lifestyle behaviours: a prospective controlled trial of rehabilitation inpatients. Patient Educ Couns 2011; 82: 186–192. 20554148 10.1016/j.pec.2010.04.031

[8] Jenny NYY Fai TS Evaluating the effectiveness of an interactive multimedia computer-based patient education program in cardiac rehabilitation. Occup Therapy J Res 2001; 214: 260–275.

[9] Mittag O China C Hoberg E, et al Outcomes of cardiac rehabilitation with versus without a follow-up intervention rendered by telephone (Luebeck follow-up trial): overall and gender-specific effects. Int J Rehabil Res 2006; 29: 295–302. 17106345 10.1097/MRR.0b013e328010ba9a

[10] Kay RH LeSage A. Examining the benefits and challenges of using audience response systems: a review of the literature. Comput Educ 2009; 53: 819–827.

[11] Beatty ID Gerace WJ Dufresne RJ. Designing effective questions for classroom response system teaching. Am J Phys 2005; 74: 31–39.

[12] Cain J Black EP Rohr J. An audience response system strategy to improve student motivation, attention, and feedback. Am J Pharm Educ 2009; 73: 21. 19513159 10.5688/aj730221 PMC2690899

[13] Fitzpatrick KA Finn KE Campisi J. Effect of personal response systems on student perception and academic performance in courses in a health sciences curriculum. Adv Physiol Educ 2011; 35: 280–289. 21908838 10.1152/advan.00036.2011

[14] Polat U Arpacı A Demir S, et al Evaluation of quality of life and anxiety and depression levels in patients receiving chemotherapy for colorectal cancer: impact of patient education before treatment initiation. J Gastrointestinal Oncol 2014; 54: 270. 10.3978/j.issn.2078-6891.2014.034 PMC4110488 25083300

[15] Kendel F Wirtz M Dunkel A, et al Screening for depression: Rasch analysis of the dimensional structure of the PHQ-9 and the HADS-D. J Affect Disord 2010; 1223: 241–246. 10.1016/j.jad.2009.07.004 19665236

[16] Ware J Jr Kosinski M Keller SD. A 12-item short-form health survey: construction of scales and preliminary tests of reliability and validity. Med Care 1996; 343: 220–233. 10.1097/00005650-199603000-00003 8628042

[17] Tregonning AM Doherty DA Hornbuckle J, et al The audience response system and knowledge gain: a prospective study. Med Teach 2012; 34: 269–274. 22455719 10.3109/0142159X.2012.660218

[18] Rubio EI Bassignani MJ White MA, et al Effect of an audience response system on resident learning and retention of lecture material. AJR Am J Roentgenol 2008; 190: 319–322. 10.2214/AJR.07.3038 18492872

[19] Miller M Hartung SQ. Evidence-based clicker use: audience response systems for rehabilitation nurses. Rehab Nurs 2012; 373: 151–159. 10.1002/RNJ.00041 22549633

[20] Kureshi F Kennedy KF Jones PG, et al Association between cardiac rehabilitation participation and health status outcomes after acute myocardial infarction. JAMA Cardiol 2016; 1: 980–988. 27760269 10.1001/jamacardio.2016.3458 PMC5482268

[21] Salzwedel A Heidler MD Haubold K, et al Prevalence of mild cognitive impairment in employable patients after acute coronary event in cardiac rehabilitation. Vasc Health Risk Manag 2017; 13: 55–60. 28260915 10.2147/VHRM.S121086 PMC5328136

