# Supplementary material for: Interactive patient education via an audience response system in cardiac rehabilitation
Source: SAGE Open Med. 2020 Aug 25;8:2050312120942118. doi: 10.1177/2050312120942118 (PMC7453440; doi:10.1177/2050312120942118)
Supplement: all_questionnaires_150 – Supplemental material for Interactive patient education via an audience response system in cardiac rehabilitation [file all_questionnaires_150.docx]

**Appendix: Questionnaires**

German versions

HADS


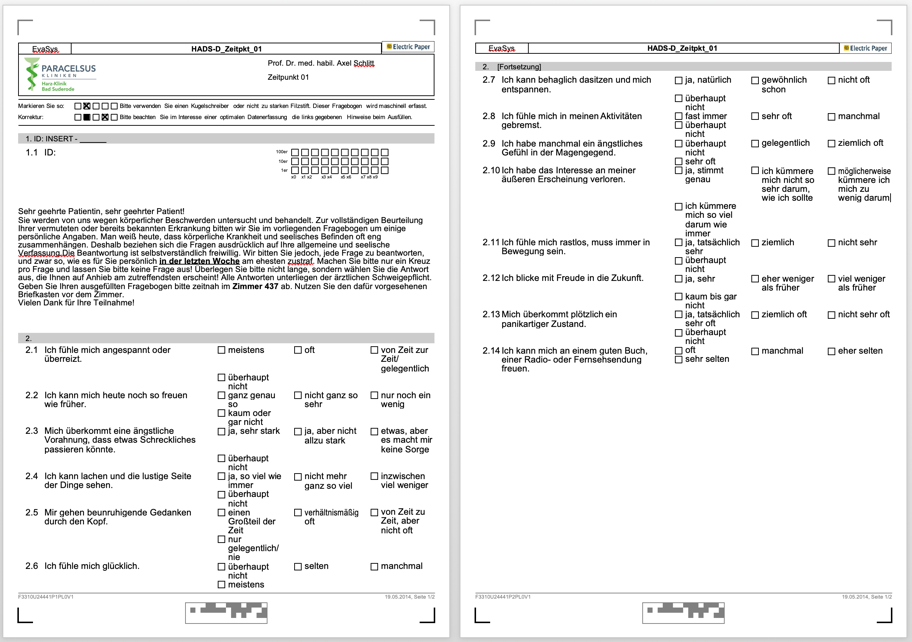


SF-12


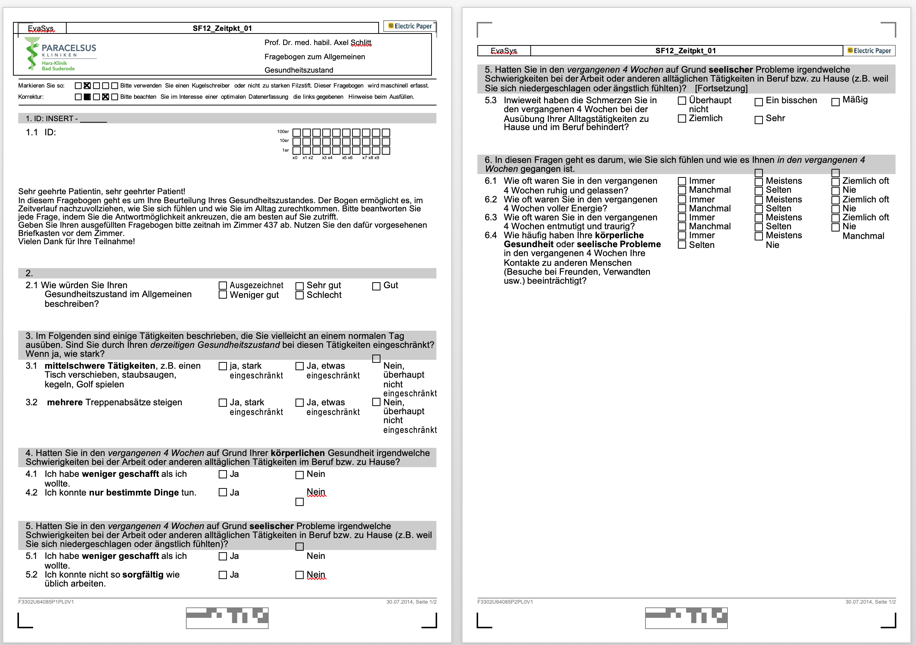


International/english versions

HADS


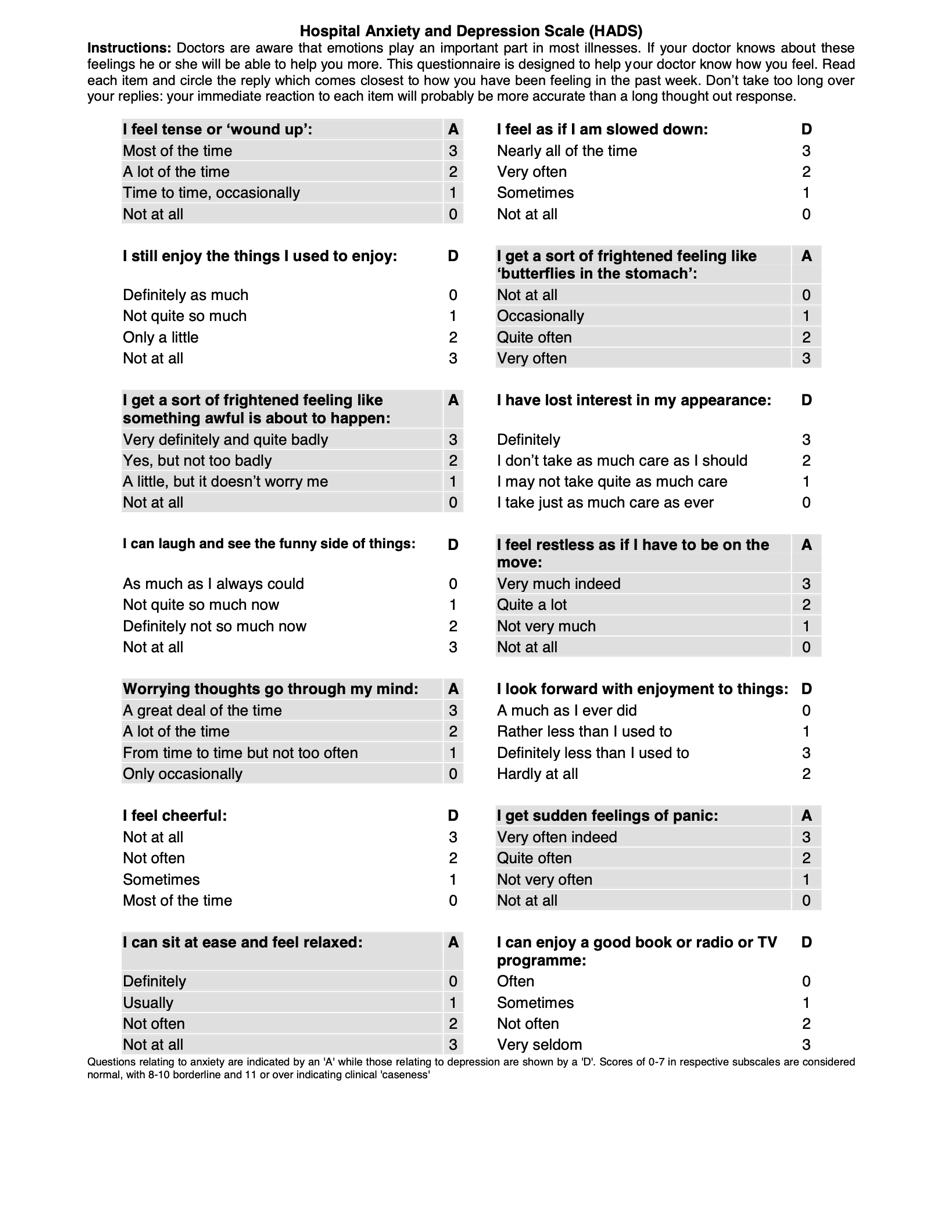


Source:

<https://studylib.net/doc/7854317/hospital-anxiety-and-depression-scale--hads->

SF-12


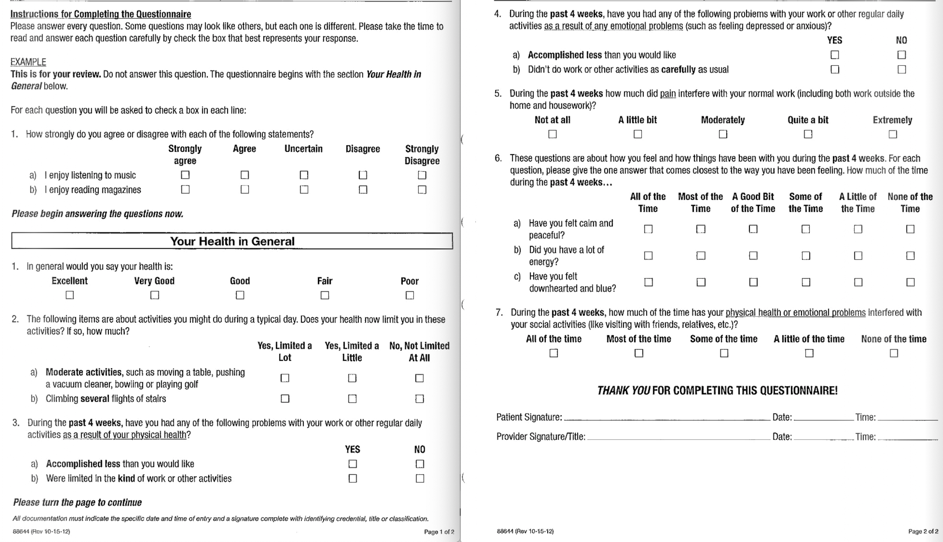


Source: <file:///Users/LisaHielscher/Library/Mobile%20Documents/com~apple~CloudDocs/ZNA%20QM%20Lisa/SAGE%20Open%20Medicine/Fragebögen/SF-12/SF12%20Health%20Survey%20.webarchive>
