# Supplementary material for: Interactive patient education via an audience response system in cardiac rehabilitation
Source: SAGE Open Med. 2020 Aug 25;8:2050312120942118. doi: 10.1177/2050312120942118 (PMC7453440; doi:10.1177/2050312120942118)
Supplement: Supp_material_Bestion_et_al._review – Supplemental material for Interactive patient education via an audience response system in cardiac rehabilitation [file Supp_material_Bestion_et_al._review.pdf]

SUPPLEMENTAL MATERIALS

GNS561 acts as a potent anti-fibrotic and pro-fibrolytic agent in liver fibrosis through TGF-β1 inhibition

Eloïne Bestion[1,2], Zuzana Macek Jilkova[3, 4, 5], Jean-Louis Mège[2], Marie Novello[1], Keerthi Kurma[3, 4, 5], Seyedeh Tayebeh Ahmad Pour[3, 4, 5], Gilles Lalmanach[6, 7], Lise Vanderlynden[6, 7], Lionel Fizanne[8], Firas Bassissi[1], Madani Rachid[1], Jennifer Tracz[1], Jérôme Boursier[8], Jérôme Courcambeck[1], Cindy Serdjebi[1], Christelle Ansaldi[1], Thomas Decaens[3, 4, 5], Philippe Halfon[1#], Sonia Brun[1#]

Address correspondence to: Dr Sonia BRUN and Dr Philippe HALFON, Address: Genoscience Pharma, 10 Rue d'Iéna, 13006 Marseille, France, Tel: +33 4 91 26 99 50, E-mail: brun.sonia@hotmail.fr and phalfon@genosciencepharma.com

Table of contents

Supplementary Materials and Methods 2
Figure S1. GNS561 decreases cell viability in LX-2 cells 11
Figure S2. GNS561 doesn't induce a significant change of reactive oxidative species levels in LX-2 cells 12
Figure S3. GNS561 decreases α-SMA and COL1A1 mRNA level in human primary HSC 13
Figure S4. GNS561 decreases α-SMA and COL1A1 mRNA level in LX-2 cells cultured in normal medium condition 14
Table S1. Primer sequences for real-time PCR of cDNA from frozen rat liver tissue samples 15
References 16

Supplementary Materials and Methods

Reagents and antibodies

Bafilomycin A1 (Baf), ammonium chloride (NH_4Cl), cOmplete™ Protease Inhibitor Cocktail, E-64, CA-074, Pepstatin A, Direct Red 80 (Sirius red), Mowiol and diethylnitrosamine (DEN) were obtained from Sigma-Aldrich (St Louis, MO, USA). Mammalian Cell Lysis Buffer (GE Healthcare, Chicago, IL, USA) was used. The fluorogenic substrates Z-Phe-Arg-7-amido-4-methylcoumarin (Sigma-Aldrich), Z-Arg-Arg-7-amido-4-methylcoumarin (Sigma-Aldrich) and (7-methoxycoumarin-4-yl)acetyl-Gly-Lys-Pro-Ile-Leu-Phe-Phe-Arg-Leu-Lys(Dnp)-D-Arg-NH_2 (Sigma-Aldrich) were used. Recombinant human transforming growth factor-beta 1 (TGF-β1) was purchased from R and D Systems (Minneapolis, MN, USA).

For western blotting assays, rabbit anti-light chain 3 phosphatidylethanolamine conjugate (LC3-II) (Sigma-Aldrich), mouse anti-alpha smooth muscle actin (α-SMA) (Sigma-Aldrich), rabbit anti-collagen type I alpha 1 chain (COL1A1) (Cell Signaling Technology, Danvers, MA, USA), mouse anti-glyceraldehyde-3-phosphate dehydrogenase (GAPDH) (Abnova, Taipei, Neihu, Taïwan), rabbit anti-Poly(ADP-ribose) polymerase (PARP) (GeneTex, Irvine, CA, USA), goat anti-cathepsin D (CTSD) (Santa Cruz Biotechnology, Dallas, TX, USA), rabbit anti-cathepsin B (CTSB) (Santa Cruz Biotechnology), goat anti-cathepsin L (CTSL) (Santa Cruz Biotechnology), rabbit anti-latency-associated peptide (LAP) (R and D Systems), rabbit anti-Smad2 (D43B4) (Cell Signaling Technology), rabbit anti-Smad3 (C67H9) (Cell Signaling Technology), rabbit anti-phospho-Smad2 (Ser465/Ser467) (E8F3R) (Cell Signaling Technology), rabbit anti-phospho-Smad3 (Ser423/425) (C25A9) (Cell Signaling Technology), rabbit anti-phospho-p44/42 MAPK (Erk1/2) (Thr202/Tyr204) (Cell Signaling Technology), rabbit anti-p44/42 MAPK (Erk1/2) (Cell Signaling Technology), donkey anti-goat antibody (Santa Cruz Biotechnology), goat anti-mouse (Jackson ImmunoResearch Europe, UK) and goat anti-rabbit (Jackson ImmunoResearch or Millipore, Burlington, MA, USA) antibodies were used.

For immunofluorescence assay, Smad4 (D3R4N) XP® Rabbit mAb (Cell Signaling Technology), goat anti-rabbit IgG (H+L) Highly Cross-Adsorbed Secondary Antibody Alexa Fluor 488 (Invitrogen, Carlsbad, CA, USA) and Hoechst 33342 Solution (ThermoFisher, Walth Waltham, MA) were used.

For immunohistochemistry experiment, rabbit anti-α-SMA monoclonal antibody (D4K9N) (Cell Signaling Technology) and goat anti-rabbit AF647 secondary antibody (Invitrogen) were used.

Study of LX-2 activation through TGF-β1

To study the effect of GNS561 on TGF-β1 stimulation in LX-2 cells, cells were incubated for 24 h in medium containing 1% fetal bovine serum (FSB). GNS561 was then added at increasing concentrations (0.75-6 µM) 2 h before addition of 5 ng/µL TGF-β1. Cell lysates were prepared 24 h after the addition of GNS561 and analyzed by quantitative real-time PCR and western blotting.

Study of primary human hepatic stellate cells activation through TGF-β1

To study the effect of GNS561 on TGF-β1 stimulation in human hepatic stellate cells (HSC), cells were first plated during 24 h in medium containing 10% FBS to allow their correct attachment on plate surface. In a second time, cells were cultured in medium without FBS for 24 h. Then, cells were treated with increasing concentrations of GNS561 (0.75-1.5 µM) 2 h before addition of 5 ng/mL TGF-β1. Cells were harvest 24 h after the addition of GNS561 and analyzed by quantitative real-time PCR.

Cell viability assay of LX-2 cells and primary human HSC

LX-2 cell and primary human HSC viability was assessed using the CellTiter-Glo Luminescent Cell Viability Assay following the manufacturer's protocol (Promega, Madison, WI, USA). Briefly, cells were plated in a 96-well tissue culture plate (6,000 cells per well for LX-2 cells and 1,000 cells per well for

primary human HSC) in 90 µL of medium. Twenty-four hours after plating, cells were treated with 10 µL of increasing concentrations of GNS561 (0.75-9 µM for LX-2 cells and (0.003-100 µM) for primary human HSC) or with GNS561 vehicle and were incubated for 24 h and 72 h. At the end of the treatment, 100 µL of CellTiter-Glo solution was added to each well; cells were briefly shaken and then were incubated at room temperature for 10 min to allow stabilization of the luminescent signal. The luminescence was recorded using an Infinite F200 Pro plate reader (Tecan, Männedorf, Switzerland) and cell viability was expressed as a percentage of the values obtained from the negative control cells (vehicle treated cells). The half-maximal inhibitory concentration (IC_{50}) was evaluated using a nonlinear regression curve in GraphPad Prism 7 (GraphPad Software, La Jolla, CA, USA). Each concentration was tested in triplicate. Mean IC_{50} was calculated as the average of three independent experiments.

Caspase activity assay

The activity of caspases 3/7 was measured using the Caspase-Glo 3/7 Assay following the manufacturer's protocol (Promega). Briefly, LX-2 cells were plated in a 96-well plate (6,000 cells per well) in 90 µL of medium. Twenty-four hours after plating, cells were treated with 10 µL of GNS561 (0.75-9 µM) or GNS561 vehicle and incubated for 24 h. At the end of the treatment, 100 µL of Caspase-Glo 3/7 reagent was added to each well and cells were incubated for 1 h at room temperature. Then, luminescence was measured by an Infinite F200 Pro plate reader. Fold change of activation of caspases 3/7 was determined by comparing the luminescence in the treated groups with the luminescence observed in the negative control wells (vehicle treated cells), with the luminescence of blank wells subtracted. At each time point, in parallel with the activation of caspases 3/7, cell viability was also investigated using CellTiter-Glo Luminescent Cell Viability Assay. Each GNS561 concentration was tested in triplicate in three independent experiments.

Reactive oxygen species assay

Reactive oxygen species (ROS) were measured using ROS-Glo H2O2 Assay following the manufacturer's protocol (Promega). LX-2 cells were plated in a 96-well plate (6,000 cells per well) in 70 µL of medium. Twenty-four hours after plating, the cells were treated with 10 µL of GNS561 (0.75-9 µM) or vehicle and incubated for 24 h. Six hours before the end of treatment, 20 µL of H2O2 substrate was added to each well. At the end of the treatment, 100 µL of ROS-Glo Detection Solution was added to each well and cells were incubated for 20 min at room temperature. Then, luminescence was measured by an Infinite F200 Pro plate reader. Fold change of ROS quantity was determined by comparing the luminescence in the treated groups to the luminescence observed in the control wells (vehicle treated cells). At each time point, in parallel, cell viability was also investigated using the CellTiter-Glo Luminescent Cell Viability Assay. Each GNS561 concentration was tested in triplicate in three independent experiments.

Lysosomotropism-mediated death study

The effect of lysosomotropism on anti-fibrotic activity was assessed as previously described[1]. LX-2 cells were plated in a 96-well plate (6,000 cells per well) in 80 µL of medium. Twenty-four hours after plating, cells were pre-treated with 10 µL of Baf (100 nM) or NH_4Cl (10 mM) for 2 h and then treated with GNS561 (1.5-9 µM) or vehicle and incubated for 24 h. At the end of the treatment, cell viability was assessed using the CellTiter-Glo Assay. Cell viability was expressed as a percentage of the values obtained from the control cells (vehicle treated cells). Each condition was tested in triplicate, and three independent experiments were performed.

Autophagy assay

The autophagy pathway was studied as performed previously[1]. Twenty-four hours after LX-2 cell plating, the cells were treated with GNS561 (1.5-6 µM) for 24 h. Treatment with vehicle was used as a

baseline for autophagic flux control. In specified conditions, Baf (100 nM) was added for the last 2 h of treatment.

Western blotting

In brief, cells were lysed with Mammalian Cell Lysis Buffer. cOmplete™ Protease Inhibitor Cocktail was added extemporaneously to the lysis buffer. Ten to twenty micrograms of protein from each sample was separated on a 15% or 4-15% SDS-PAGE gel, transferred to a PVDF membrane, and blotted with antibodies against LC3-II (1:3,000), PARP (1:1,500), α-SMA (1:2,000), COL1A1 (1:1,000), LAP (2 μg/mL), Smad2 (1:1,000), Smad3 (1:1,000), phospho-SMAD2 (1:1,000), phospho-Smad3 (1:1,000), phospho-p44/42 MAPK (Erk 1/2) (1:1,000), p44/42 MAPK (Erk 1/2) (1:1,000), CSTB (1:200), CSTD (1:200) or CSTL (1:200). For all blots, GAPDH immunoblotting (1:5,000) was used as a loading control. The autophagic flux was calculated as the ratio between the LC3-II level normalized against GAPDH level (Norm LC3-II) with Baf and without Baf. The LAP cleavage ratio was determined as the ratio between the LAP level and the pro-TGF-β1 level for each condition in comparison with the ratio obtained for the condition without GNS561 treatment. The α-SMA and COL1A1 fold changes were calculated as the ratio between α-SMA or COL1A1 level normalized against GAPDH level for each condition and α-SMA or COL1A1 level normalized by GAPDH level for the untreated condition (neither GNS561 nor TGF-β1 stimulation). The p-Smad/Smad ratio was determined as the ratio between the p-Smad and Smad level for each condition in comparison with the ratio obtained for the untreated condition (neither GNS561 nor TGF-β1 stimulation). The p-Erk1/2/Erk1/2 ratio was determined as the ratio between the p-Erk1/2 and Erk1/2 level for each condition in comparison with the ratio obtained for the untreated condition (neither GNS561 nor TGF-β1 stimulation). All the experiments were repeated at least three times. Representative autoradiograms are shown.

Cathepsin activity assay in cellular lysates

Twenty-four hours after LX-2 cell plating, the cells were treated with GNS561 (1.5-9 µM) for 24 h. Treatment with vehicle was used as a baseline for cathepsin activity control. Cell lysates (1 µg of total protein) were pre-incubated with acetate buffer (0.1 M sodium acetate, pH 5.5, 10 mM DTT, 2 mM EDTA, and 0.01% Brij35) or with citrate buffer (0.1 M sodium citrate, pH 4.0, 2 mM EDTA, 0.01% Brij35) prior to measurement of the respective CTSB/L (including both cathepsins B and L) and CTSB activities and CTSD activity. The peptidase activity of CTSB, CTSB/L and CTSD were determined fluorometrically with a fluorescence reader (Gemini spectrofluorometer, Molecular Devices, San José, CA, USA) using respectively, the synthetic substrates Z-Arg-Arg-7-amido-4-methylcoumarin (excitation wavelength: 350 nm; emission wavelength: 460 nm), Z-Phe-Arg-7-amido-4-methylcoumarin (excitation wavelength: 350 nm; emission wavelength: 460 nm) and methoxycoumarin-4-yl)acetyl-Gly-Lys-Pro-Ile-Leu-Phe-Phe-Arg-Leu-Lys(Dnp)-D-Arg-NH_2 (excitation wavelength: 325 nm; emission wavelength: 390 nm). The synthetic protease inhibitors E-64 (inhibitor of both CTSB/L), CA-074 (specific inhibitor of CTSB) and Pepstatin A (inhibitor of CTSD) were used as controls to confirm the detection of specific activities. Slopes of the enzymatic activities were calculated with the software SoftMax Pro (Molecular Devices). For each experiment and tested condition, fold change of the cathepsin activity was determined by comparing the slope of the enzymatic activity in treated conditions to the slope of the enzymatic activity in the vehicle condition. Three independent experiments were performed.

Immunocytochemistry assay

4.5×10^4 LX-2 cells were plated on glass coverslips, placed in 24-well dishes containing 1% FBS and incubated for 24 hours at 37°C. Cells were then pre-treated during 2 h by GNS561 in increasing doses followed by 5 ng/mL TGF-β treatment. 24 h after GNS561 treatment, cells were fixed in 3% paraformaldehyde in phosphate-buffered saline (PBS) for 20 minutes at 4°C. Blocking buffer (1X PBS, 5% FBS) was then added for 60 minutes at room temperature. 5 minutes before the end of blocking step, cells were permeabilized in 0.3% Triton X-100. Then, the cells were incubated with primary

antibodies against Smad4 (1/500) and with Hoechst 33342 Solution (1 µg/mL) for nuclei staining during 1 h at room temperature. Alexa Fluor 488 secondary antibody for Smad4 labelling was then applied in PBS containing 5% FBS, for 30 minutes at room temperature. Cells were mounted between slide and slip cover with Mowiol diluted 1:2 in PBS. Image acquisition was performed 24 h after labelling on a LSM 800 Airyscan confocal microscope (Carl Zeiss, Oberkochen, Germany) and collected by Zen 3.0 (Blue Edition) software (Carl Zeiss). Three independent experiments were performed.

Rat model and treatment groups

Fourteen 6-week-old Fischer 344 male rats (Charles River, Wilmington, MA, USA) were housed in the Plateforme de Haute Technologie Animale animal facility (Jean Roget, University of Grenoble-Alpes, France). Rats were kept in individually ventilated cage (IVC) systems at constant temperature and humidity with 2-3 animals in each cage having free access to food (standard diet) and water during the entire study period. All rats were treated weekly with intra-peritoneal injections of 50 mg/kg of diethylnitrosamine (DEN) (Sigma-Aldrich), which were diluted in olive oil to obtain a cirrhotic liver with hepatocellular carcinoma after 14 weeks[2]. Rats were randomized into 2 groups (n=7/group) and treated over six weeks by daily oral gavages of GNS561 (15 mg/kg of GNS561, GNS561 group) or of vehicle (control group). The nutritional state was monitored by daily weighing of the rats. The food intake per cage was monitored during the last 6 weeks of the experiment. Food was withheld for 3-4 h before the animals were sacrificed. After six weeks of treatment treatment with GNS561 or vehicle, animals were anesthetized with isoflurane and euthanized with vena cava blood sampling.

All animals received humane care in accordance with the Guidelines on the Humane Treatment of Laboratory Animals (Directive 2010/63/EU), and experiments were approved by the animal Ethics Committee: GIN Ethics Committee n°004.

Collagen and α-SMA detection in rat samples

Collagen was detected in paraffin-embedded sections with Direct Red 80 stain solution and the staining was subsequently quantified by MetaMorph software (Molecular Devices) in 10 randomly selected fields/section (10× magnification). α-SMA staining was detected on paraffin-embedded sections by α-SMA (D4K9N) rabbit monoclonal antibody followed by goat anti-rabbit AF647 secondary antibody. Images were captured using an ApoTome microscope (Carl Zeissequipped with a camera AxioCam MRm and collected by AxioVision software (Carl Zeiss). Positive areas were quantified using ImageJ software (NIH, Bethesda, MD, USA) on 15 randomly selected fields/section (10× magnification). All analyses were performed in a double blinded manner.

Gene expression analysis by quantitative real-time PCR

Total RNA was isolated from LX-2 or primary human HSC cells using the RNeasy Mini kit (Qiagen, Valencia, CA, USA) and was reverse-transcribed into complementary DNA (cDNA) using the Transcriptor First Strand cDNA Synthesis Kit (Roche, Bâle, Switzerland). Amplification reactions were performed in a total volume of 20 µL by using the Cobas z 480 PCR system (Roche) and TB Green qPCR Premix Ex Taq (Tli RNaseH Plus) (Ozyme, Saint-Cyr-l'École, France) with the following primers obtained from Qiagen, *ASMA* (catalogue number PPH01300B), *COL1A1* (PPH01299F), *TGFB1* (PPH00508A), *MMP2* (PPH00151B), *MMP9* (PPH00152E), *TIMP1* (PPH00771C), *TIMP3* (PPH00762B) and *B2M* (PPH01094E). Data were analyzed with the LightCycler 480 SW 1.5 software (Roche) and fold changes were calculated as the relative expression of fibrogenic genes after normalizing to B2M used as housekeeping gene compared with the untreated condition (neither GNS561 nor TGF-β1 stimulation). A melting curve analysis was done after amplification to verify the accuracy of the amplicon. Every analysis was done in duplicate and three independent experiments were performed.

Total RNA from frozen rat liver tissue samples was extracted using the RNeasy Mini Kit (Qiagen). Reverse transcription was realized with iScript™ gDNA clear cDNA Synthesis Kit (Bio-Rad, Hercules, CA,

USA), and amplification reactions were performed with the primers listed in Table S1 in a total volume of 10 µL by using a Thermocycler sequence detector (Bio-Rad CFX96) with the iTaq Universal SYBR Green Supermix qPCR kit (Bio-Rad). Primers were designed with the Primer 3 command line program (version 4.0.0) and verified with BLAST. Oligonucleotide sequences were synthetized by Eurofins Genomics in 0.01 µmol scale, with a salt free level of purification. Data were analyzed with CFX Manager Software (Bio-Rad) and fold changes were calculated as the relative expression of fibrogenic genes after normalizing to GADPH (used as housekeeping gene) compared with the control group. A melting curve analysis was done after amplification to verify the accuracy of the amplicon. Every analysis was done in duplicate.

Figure S1. GNS561 decreases cell viability in LX-2 cells

LX-2 cells were exposed to different concentrations of GNS561 for 72 h. Cell viability was detected using the CellTiter-Glo viability assay. The data are presented as the mean values + SEM of three experiments.

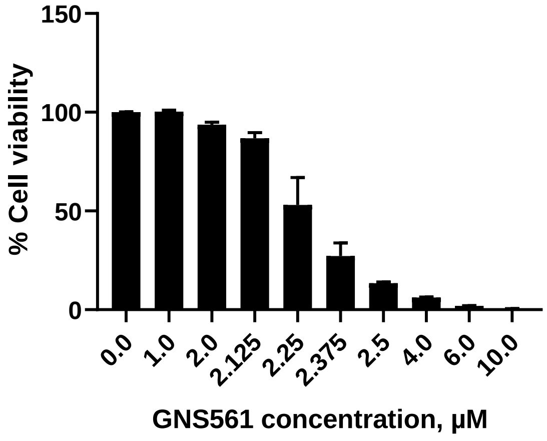

Figure S2. GNS561 doesn't induce a significant change of reactive oxidative species levels in LX-2 cells

Fold change of reactive oxidative species (ROS) and cell viability percent after 24 h of GNS561 treatment against vehicle condition were measured using the ROS-Glo H2O2 assay and the CellTiter-Glo viability assay respectively. Data represent the mean values + SEM of three experiments.

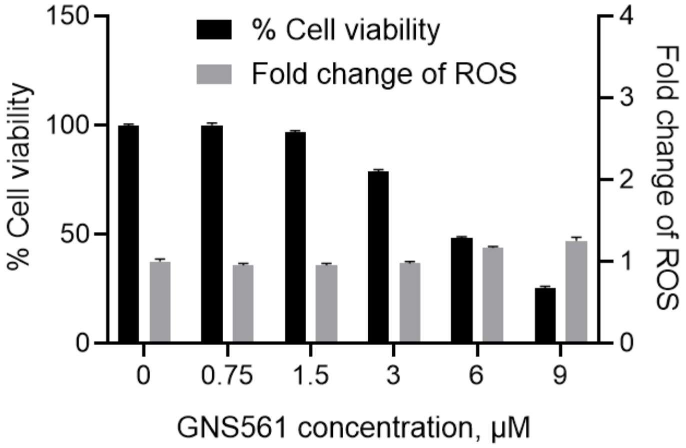

Figure S3. GNS561 decreases α-SMA and COL1A1 mRNA level in human primary HSC

Cell lysates were prepared after both GNS561 treatment (24h) and transforming growth factor-beta 1 (TGF-β1) stimulation (22h, 5 ng/µl) and analyzed by real-time PCR. mRNA fold change of alpha smooth muscle actin (α-SMA) (a) and collagen type I alpha 1 chain (COL1A1) (b) were measured in comparison with the untreated condition (neither GNS561 nor TGF-β1 stimulation). The data are presented as median values of three separate experiments surrounded by upper and lower confidence limits (95%).

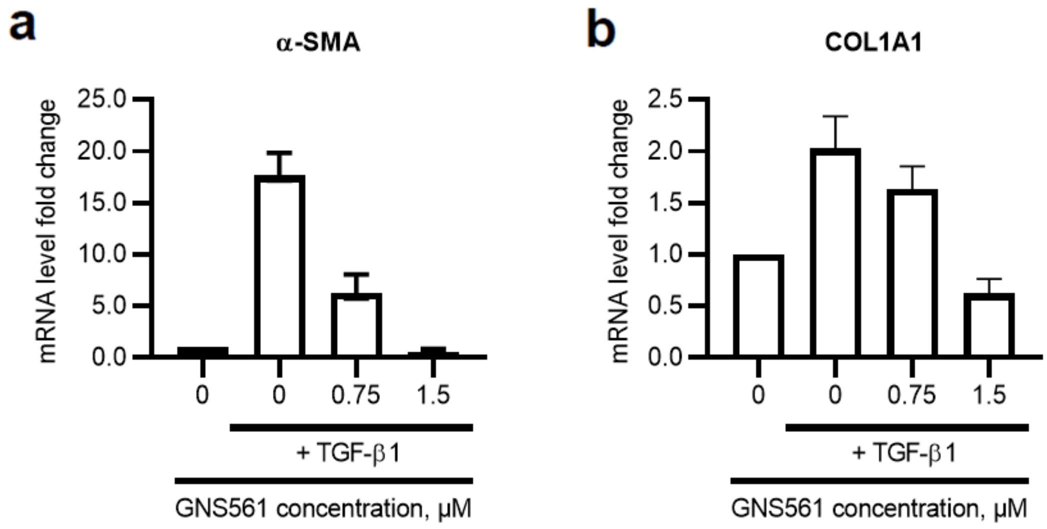

Figure S4. GNS561 decreases α-SMA and COL1A1 mRNA level in LX-2 cells cultured in normal medium condition

Cell lysates were prepared after GNS561 treatment (24h) and analyzed by real-time PCR. mRNA fold change of alpha smooth muscle actin (α-SMA) (a) and collagen type I alpha 1 chain (COL1A1) (b) were measured in comparison with the untreated condition (no GNS561 treatment). The data are presented as median values of three separate experiments surrounded by upper and lower confidence limits (95%).

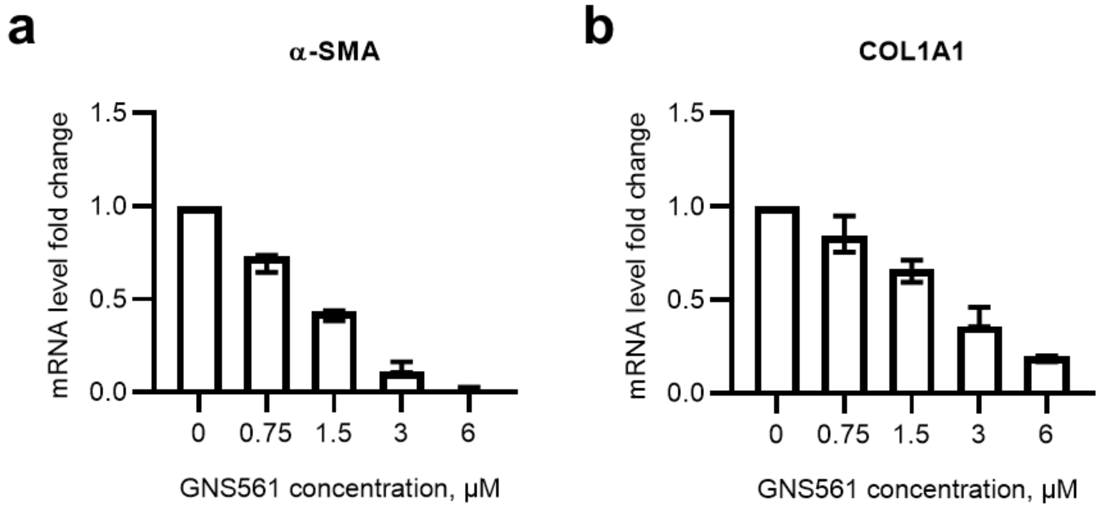

Table S1. Primer sequences for real-time PCR of cDNA from frozen rat liver tissue samples

Gene	Reverse Sequence (5’-3’)	Forward Sequence (5’-3’)
Col1a1	CTTCTGGGCAGAAAGGACAG	GCCAAGAAGACATCCCTGAA
Asma	CATCTCCAGAGTCCAGCACA	ACTGGGACGACATGGAAAAG
Tgfb1	TGGGACTGATCCCATTGATT	ATACGCCTGAGTAGCTGTCT
Timp1	TGGCTGAACAGGGAAACACT	CAGCAAAAGGCCTTCGTAAA
Mmp2	GGGTTTCTTCTGGCTCAGG	TCTGGCTATCCACAAGACTGG
Mmp9	GGAAAAGGAAGGAGGGTACG	CCACTCAGGGCCTTCAGAC
Gapdh	TTCAGCTCTGGGATGACCTT	CTCATGACCACAGTCCATGC

References

1. Brun S, Bassissi F, Serdjebi C, et al. GNS561, a new lysosomotropic small molecule, for the treatment of intrahepatic cholangiocarcinoma. *Invest New Drugs* 2019 2019/02/20. DOI: 10.1007/s10637-019-00741-3.
2. Jilkova ZM, Kuyucu AZ, Kurma K, et al. Combination of AKT inhibitor ARQ 092 and sorafenib potentiates inhibition of tumor progression in cirrhotic rat model of hepatocellular carcinoma. *Oncotarget* 2018; 9: 11145-11158. 2018/03/16. DOI: 10.18632/oncotarget.24298.
